# Transcriptomic Profiling of iPS Cell-Derived Hepatocyte-like Cells Reveals Their Close Similarity to Primary Liver Hepatocytes

**DOI:** 10.3390/cells14120925

**Published:** 2025-06-18

**Authors:** Saqlain Suleman, Sharmin Alhaque, Andrew Guo, Aaron Zhang, Serena Fawaz, Stefany Perera, Mohammad S. Khalifa, Hassan Rashidi, David C. Hay, Michael Themis

**Affiliations:** 1 School of Life Sciences, Faculty of Science and Engineering, Angila Ruskin University, Cambridge CB1 2PU, UK; 2 Division of Biosciences, Department of Life Sciences, College of Health, Medicine & Life Sciences, Brunel University of London, London UB8 3PH, UK; 3 Testavec Ltd., Queensgate House, Maidenhead SL6 8BD, UK; 4 Biomavericks, 64 Nile Street, London N1 7SR, UK; 5 Centre for Regenerative Medicine, The University of Edinburgh, Edinburgh EH16 4UU, UK; 6 UCL Great Ormond Street Institute of Child Health, University College London, London WC1N 1EH, UK; 7 Division of Infection and Immunity, University College London, London WC1E 6BT, UK

**Keywords:** liver, transcriptomic, stem cells, differentiation

## Abstract

Human-induced pluripotent stem cell (iPSC)-derived hepatocyte-like cells (HLCs) have been shown to be useful for the development of cell-based regenerative strategies and for modelling drug discovery. However, stem cell-derived HLCs are not identical in nature to primary human hepatocytes (PHHs), which could affect the cell phenotype and, potentially, model reliability. Therefore, we employed the in-depth gene expression profiling of HLCs and other important and relevant cell types, which led to the identification of clear similarities and differences between them at the transcriptional level. Through gene set enrichment analysis, we identified that genes that are critical for immune signalling pathways become downregulated upon HLC differentiation. Our analysis also found that TAV.HLCs exhibit a mild gene signature characteristic of acute lymphoblastic leukaemia, but not other selected cancers. Importantly, HLCs present significant similarity to PHHs, making them genuinely valuable for modelling human liver biology in vitro and for the development of prototype cell-based therapies for pre-clinical testing.

## 1. Introduction

Liver disease is a leading cause of mortality and morbidity worldwide, accounting for approximately 2 million deaths annually [1]. While liver transplantation is widely adopted as a treatment for end-stage liver disease, challenges persist, including the lack of acceptable donor organs, graft rejection, and the recurrence of viral hepatitis after liver transplantation. Therefore, more widely available treatments are required, which include the development of renewable cell-based therapies [2]. A variety of candidate cell types have been proposed as starting materials [3], yet it is still unclear whether these cells, when grown in vitro, exhibit similar characteristics to those of primary human hepatocytes (PHHs).

Induced pluripotent stem cells (iPSCs) offer a renewable resource to generate somatic cells, including hepatocyte-like cells (HLCs). iPSCs and their HLC derivatives have been widely used as validated models for human disease and pharmacotoxicological assessments [3,4,5,6,7,8,9,10,11,12], and they have potential for use in the correction of genetic disorders [13,14]. Although HLCs share similar features with PHHs at the transcriptomic level, differential gene expression patterns have been detected. These differences in gene expression require further examination, especially if HLCs are to be successfully deployed in disease modelling and drug development studies.

To address this issue, we used RNA-seq data for iPSCs and HLCs and aligned the sequencing output to a variety of cell types, including PHHs. Genomic alignment and expression profiling through unsupervised clustering and principal component analysis revealed that HLCs matched closely with primary hepatocytes. By cross-comparing the data, we generated differentially expressed genes and annotated them with key biological processes that are critical for data interpretation. These were then used for a carcinogenesis assessment via machine learning algorithms, which enabled us to determine gene signatures that were specific to cancer.

## 2. Materials and Methods

### 2.1. Generation of TAV.iPSC and TAV.HLC Derivatives

A human iPSC line (JHUP106i, hereafter denoted as TAV.iPSC) was cultured routinely on laminin 521 (BioLamina, Interchim, France)-coated plates in serum-free mTeSR™1 medium (STEMCELL Technologies, Cambridge, UK), as previously described [15]. Cells were monitored regularly for infection and propagated in antibiotic-free medium. Cells were characterised for markers of pluripotency and differentiation using flow cytometry before being differentiated into HLC 3D spheroid cultures (hereafter denoted as TAV.HLC), as previously described [16,17,18]. Immunostaining (against HNF4A) and q-RT PCR (against *HNF*4*A* and *ALB*) were also used to identify hepatocyte markers, as previously described [15,18].

Cyp3A activity was assessed using 50 uM of Luciferin-PFBE substrate (Promega, Southampton, UK), which was incubated with 3D hepatospheres in HepatoZYME medium supplemented with 10 ng/mL of HGF. Cytochrome P450 activity was measured 24 h later using the P450-Glo assay kit (Promega), following the manufacturer’s instructions. To measure AFP and ALB secretion, supernatants were collected after 24 h and quantified using commercial ELISA kits (Alpha Diagnostics International, San Antonio, TX, USA). Data were normalised with the total protein content measured using a bicinchoninic acid (BCA) assay (Thermo Fisher Scientific, Loughborough, UK).

### 2.2. RNAseq Sample Preparation

RNA was extracted from TAV.iPSCs and TAV.HLCs (n = 2), differentiated as previously reported [16,17,18], using the RNAeasy Mini RNA Extraction Kit (Qiagen, Manchester, UK) according to the manufacturer’s instructions. RNA was treated with on-column DNAase I digestion according to the manufacturer’s instructions (Qiagen). RNA quantity and quality were evaluated using NanodropTM200c (Thermofisher Scientific, Loughborough, UK). RNA quality was assessed with the TapeStation 2200 system using TapeStation RNA ScreenTape & Reagents (Agilent, Santa Clara, CA, USA). Up to 1000 ng of total RNA per sample was applied for SureSelect Strand-Specific mRNA Library Preparation for Illumina (Agilent) and the TruSeq Stranded mRNA Library Prep Kit (Illumina, Cambridge, UK) according to the manufacturer’s instructions for library preparation. Libraries were sequenced in 150PE mode with the Illumina HiSeq System.

### 2.3. Quality Control, Alignment of Genomic DNA, and Differential Gene Analysis

The quality of RNA-seq data in FastQ files was checked by FastQC (v0.11.9). Reads were filtered, and we trimmed the raw reads with (1) Phred quality scores lower than 20, (2) a length less than 50 nt, (3) expected errors greater than 2, or (4) Ns greater than 0. Dada2 (v1.18) [16] was used to accomplish this step. The filtered and trimmed reads were then aligned to the reference genome GRCh38 (hg38; version released on 29 November 2021) using Rsubread (v3.14) [19,20]. The resulting BAM files were then annotated with genomic features by featureCounts [20]. DESeq2 (v1.34.0) was used to perform differential gene expression analysis [21]. The iPSC and HLC derivatives were denoted as TAV.iPSCs and TAV.HLCs, respectively, and were run as two biological replicates. The same method was used to perform meta-analysis on the public RNA-seq data for cross-dataset comparison. The details are summarised in Table 1.

Sample cells (TAV.iPSCs and TAV.HLCs) were used to provide RNA for the RNASeq analysis. Gene expression datasets were assigned sample numbers for downstream analysis. Sample numbers 1 and 2 are used for TAV.iPSC (JHUP106i) and TAV.HLC derivatives. Sample number 3 is used for HepG2, which represents a liver cancer cell line that can be used to determine whether the differentiated iPSCs share a certain degree of transcriptomic similarity with liver cancer. The data used from the results of iPSC differentiation in this study, using the same technology with a similar experimental design, were, therefore, considered comparable to other studies. CTRL (healthy liver tissue) and PHHs (primary human hepatocytes) serve as non-cancerous references. Samples 8–13 originate from the TCGA/TARGET database (n = 1799), which increases the total sample size to 1920 for the data analysis, markedly improving the statistical significance. Liver or leukaemia (lymphocytic or myeloid) samples were used to extract specific gene signatures for cytotoxicity assessment. Skin samples were used for a comparison with iPSCs. Samples 14–16 were obtained from teratoma, fibroblasts, or PBMCs for comparison with iPSCs or leukaemia cells, respectively.

### 2.4. Principal Component Analysis (PCA)

To investigate the overall expression profiles across different samples, we normalised the count matrix to fragments per kilobase of transcript per million mapped reads (FPKM), followed by principal component analysis (PCA), using 5000 top-ranked variable protein-coding genes. PCA was performed using samples 1–16, as described in Table 1.

### 2.5. Pathway Enrichment Analysis

Hypergeometric testing was performed by using the enricher function in the clusterProfiler package (v4.2.2) [26], aiming to assess the enrichment of hallmark signalling pathways across different samples. Genes with a fold change greater than 1 were defined as differentially expressed genes and regarded as input. Hallmark gene sets from the Molecular Signatures Database (MSigDb; v1.2.0) were used for the enrichment analysis. The enrichment analysis was performed using samples 1–16, as described in Table 1.

### 2.6. Weighted Correlation Network Analysis (WGCNA)

WGCNA was performed by using the package WGCNA_1.70-3 [27]. A minimum of 10 samples per group should normally be used for WGCNA. Briefly, we filtered out genes that were not expressed across all samples or were related to non-coding, mitochondrial, DNase/RNase, ATPase, ribosome, or unannotated open-reading frames. The filtered genes were then used for one-step network construction and module detection. We used a soft-thresholding power of 10 based on the criterion of approximate scale-free topology. WGCNA was performed using samples 1–16, as described in Table 1.

### 2.7. Phenotype Classification and Prediction

To investigate the transcriptomic similarity between the samples and the reference data with phenotypic information, we prepared training datasets for (1) a normal liver (Liver.Nor,), (2) hepatocellular carcinoma (Liver.Car), (3) normal skin (Skin.Nor), (4) melanoma (Skin.Car), (5) normal peripheral blood mononuclear cells (PBMCs), (6) acute lymphoblastic leukaemia (ALL), (7) acute myeloid leukaemia (AML), (8) teratoma, and (9) fibroblasts (Fib.) and cross-compared them with TAV.iPSC and TAV.HLC samples and reference samples (G.iPSC, G.HLC, G.PHH, G.HepG2) from Gupta et al., 2021 [22]. The package ranger was used to carry out phenotype classification and prediction.

## 3. Results

### 3.1. RNA-Seq Analysis Reveals Transcriptomic Similarity Between iPSC-Derived HLCs and PHHs

**Figure 1:**
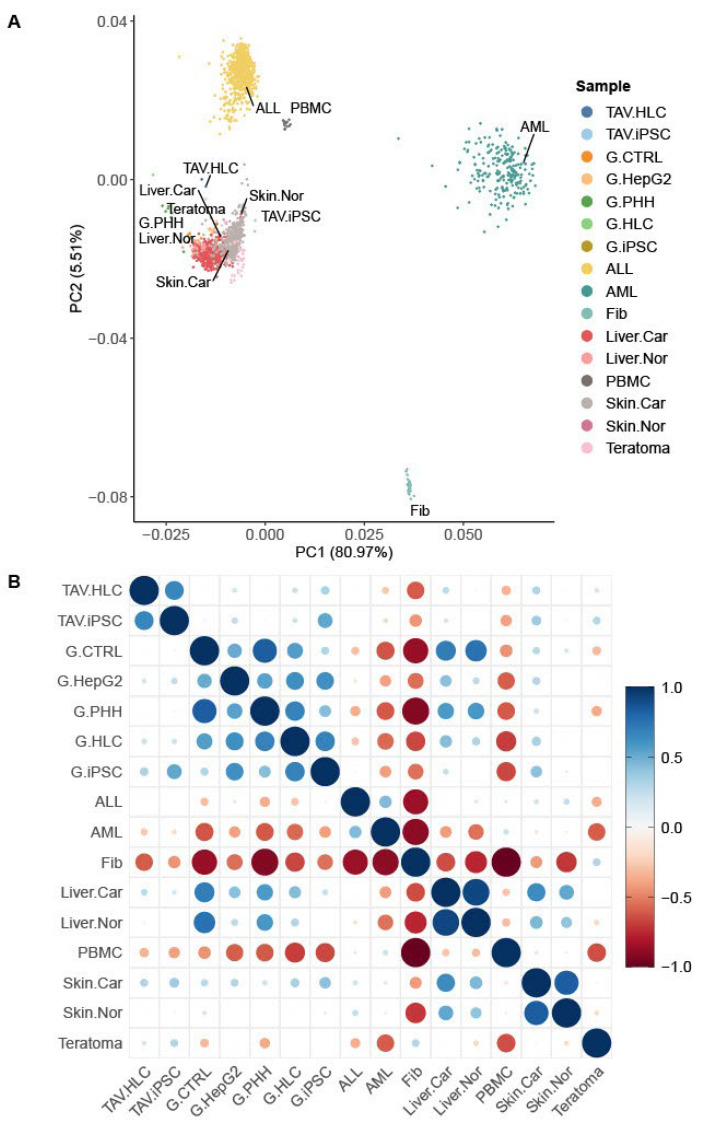
Assessment of inter- and intragroup variability. (**A**) Principal component analysis (PCA) depicting all 1920 samples along PC1 and PC2—which reveal 80.97% and 5.51% of the variability, respectively, within the expression dataset. PCA was applied to FPKM-normalised and log-transformed count data. (**B**) Pearson’s correlation matrix showing the correlation (r) values across different samples. The scale bar represents the range of the correlation coefficients (r). Dot sizes show level of statistical significance. These analyses identify close similarity between TAV.HLCs and healthy liver samples.

**Figure 2:**
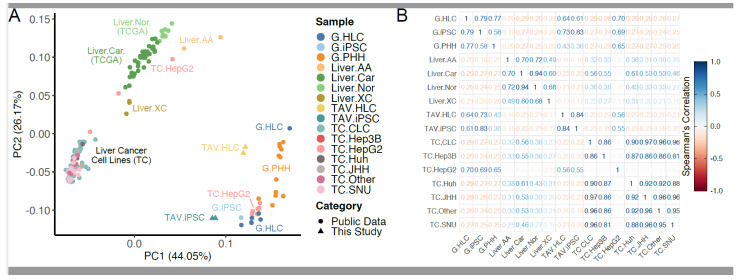
Assessment of inter- and intragroup variability in TAV.iPSC, TAV.HLC, liver, skin, and teratoma samples. (**A**) Principal component analysis depicting selected samples along PC1 and PC-which reveal 44.05% and 26.17% of the variability, respectively. (**B**) Spearman’s correlation revealing a quantitative measure of transcriptomic similarity across all liver samples. This highlights that TAV.HLCs clustered closely with PHHs and HLCs from Gupta et al., 2021 [22].

To test whether the iPSC-derived HLCs exhibit transcriptomic similarity with PHHs, principal component analysis (PCA) was used to display a comparison between the RNA-seq datasets from this study and those reported by Gupta et al., 2021 [22] (denoted by G). For this analysis, iPSC and HLC samples, termed TAV-iPSC1, TAV-iPSC2, TAV-HLC1, and TAV-HLC2, respectively, were generated in this study to investigate their transcriptomic similarities with the reference data. After averaging the results from these datasets (termed TAV.iPSC and TAV.HLC) and selecting the 5000 top-ranked variable protein-coding genes for PCA (Figure 1 and Figure 2), we found that TAV.iPSCs and TAV.HLCs appear to be aggregated with the samples from the liver, skin, and teratoma, as expected. The samples from the reference data also appeared comparable, with parallel clustering with the same sample groups. The samples from PBMC, ALL, and AML aggregated as separate clusters, suggesting that they share fewer common features with TAV.HLCs and TAV.iPSCs. The AML and ALL samples are markedly different from each other; however, the ALL samples share some similar characteristics with the PBMC samples. Fibroblasts from different tissue origins aggregated as a single cluster away from the other samples, suggesting that TAV.HLCs and TAV.iPSCs are less likely to share common features with these cells. To gain insight into the relationships between these samples, we used Pearson’s correlation test with the same dataset.

**Figure 3:**
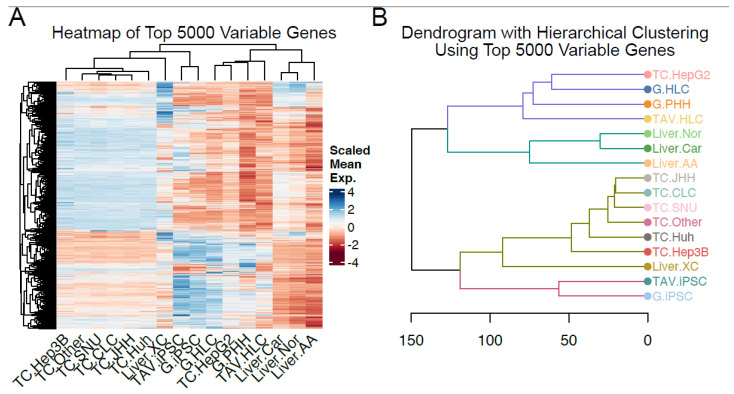
Expression profiling reveals transcriptomic similarity across liver samples. (**A**) Heatmap showing mean expression levels of the top 5000 variable genes across each sample column. (**B**) Dendrogram depicting the transcriptomic distance between the liver samples based on these 5000 variable genes. We identified similarities between the top 5000 variable gene subset of TAV.HLCs and primary hepatocytes.

By performing unsupervised hierarchical clustering using the top 5000 differentially expressed genes across each sample (averaged and grouped for clarity), we show that TAV.HLCs are clustered with G.HLCs generated by Gupta et al., 2021. [22], suggesting that the top 5000 differentially expressed genes between these samples are partially shared between these datasets (Figure 2). TAV.iPSCs cluster with skin samples, suggesting pluripotent characteristics (Figure 2). While this differs from the first PCA plot in Figure 1, this is likely due to variable features in the first two principal components. The iPSCs and HLCs from the sample data appear more closely related to G.iPSCs and G.HLCs, as expected. The PBMC/leukaemia, fibroblast, and teratoma sample are clustered separately, suggesting that TAV.iPSCs and TAV.HLCs do not share distinct features with these samples (Figure 3).

To rule out the possibility that the differentiated HLCs may share transcriptomic similarities with other tissues, we cross-compared these datasets with the ones from healthy peripheral blood mononuclear cells (PBMCs), acute lymphocytic leukaemia (ALL), acute myeloid leukaemia (AML), teratoma, and tissue-specific fibroblasts, which showed this not to be the case. TAV.HLCs can be seen across these differentially expressed genes (DEGs) to be transcriptomically similar to primary hepatocytes (Figure 2 and Figure 3).

### 3.2. Pathway Analysis Shows Downregulated Genes Characteristic of Immune-Associated Signalling Pathways in iPSCs Compared to Those in HLCs

**Figure 4:**
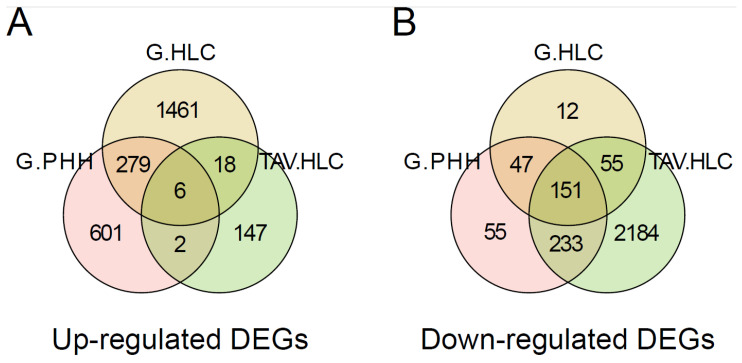
Gene expression regulation in iPSC and HLCs. Venn diagram showing the numbers of (**A**) up- or (**B**) downregulated genes shared between the PHH, G.HLC, and TAV.HLC datasets. These indicate differences between datasets, which are likely attributed to differences in differentiation protocols.

**Figure 5:**
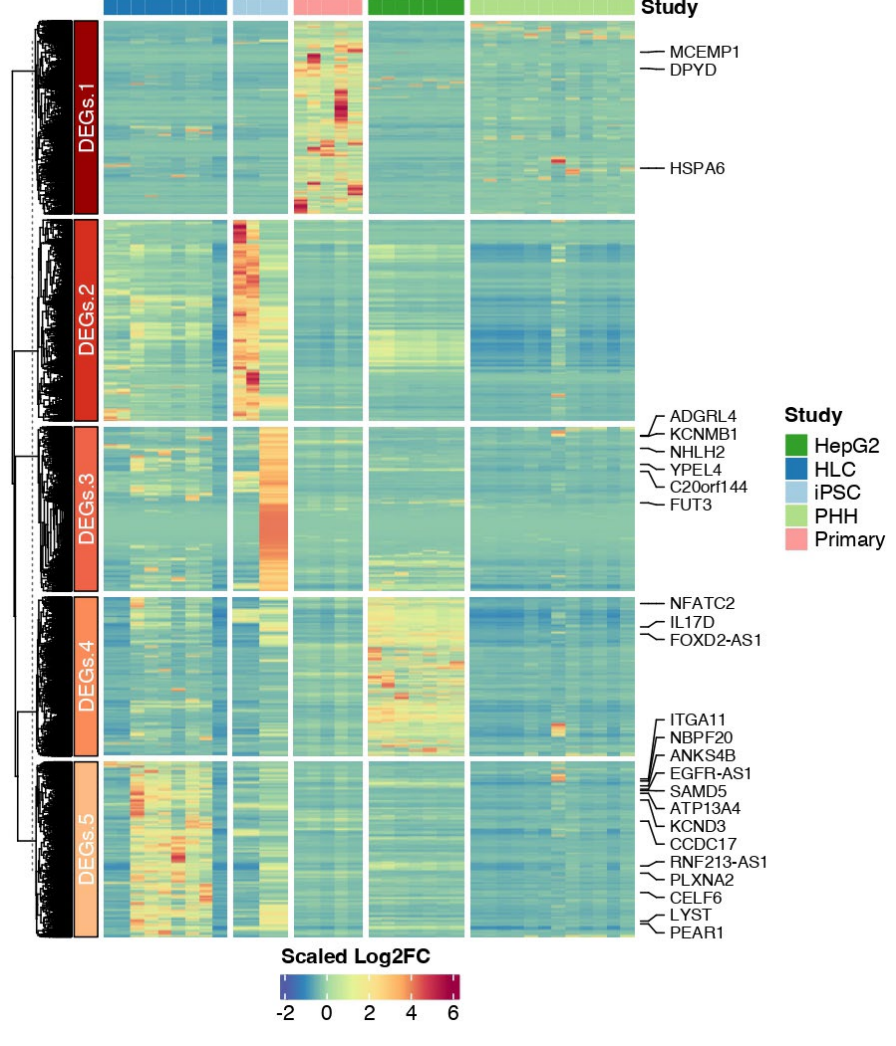
Heatmap showing scaled mean expression levels of the top DEGs from the available datasets used in this study. Expression levels in iPSCs (TAV.iPSC), HLCs (TAV.HLC), PHHs (Liver.Nor), and HepG2 (Liver.Car) across different samples. Using scatter plots of these genes, many of downregulated genes such as *HMGB*2, *BIRC*5, *ARID*3*B*, and *SOX*2, they are found to be associated with the cell cycle.

**Figure 6:**
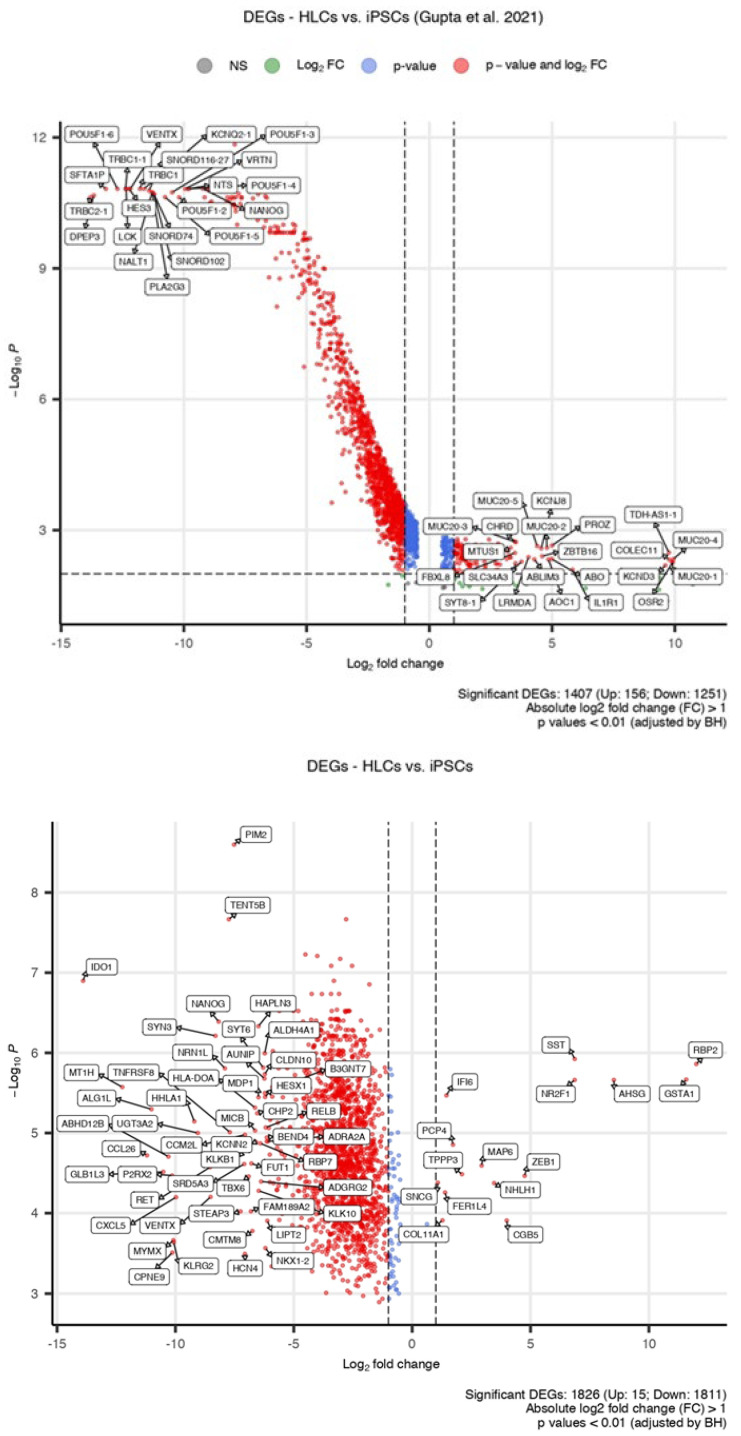
Differentially expressed genes in iPSCs and HLCs. Scatter plot showing *p* values and log2 fold change in each differentially expressed gene in G.HLCs (**top**) and TAV.HLCs (**bottom**). Upregulated (+1 fold change) and (−1 fold change) downregulated genes are shown. The most significant genes that are differentially regulated are labelled [22].

We then performed a differential analysis of genes highly expressed in iPSCs or HLCs to gain insight into their biological implications. Through cross-comparison with the reference datasets, we identified similarly up- (n = 6) or downregulated (n = 151) genes in the TAV.HLCs (Figure 4, Figure 5 and Figure 6).

**Figure 7:**
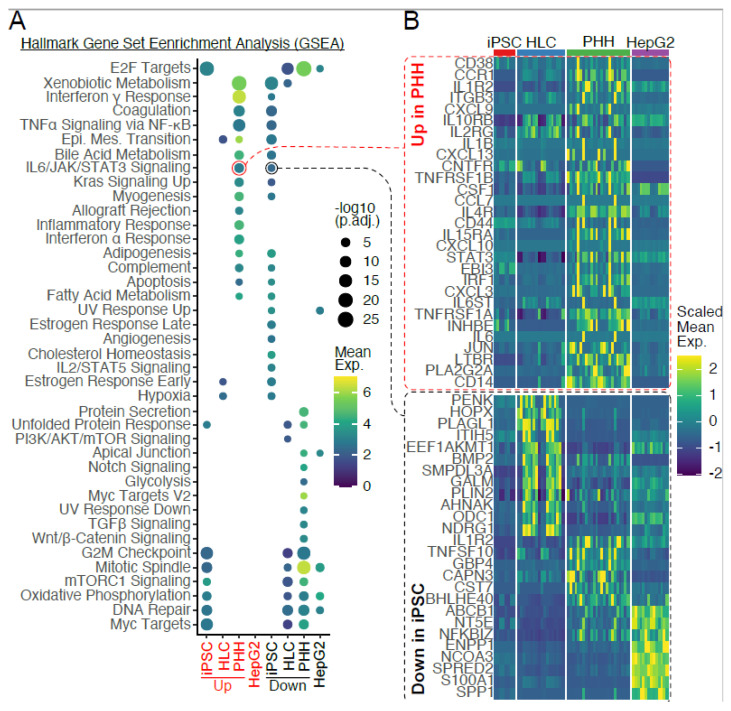
Hallmark gene set enrichment analysis highlights differentially expressed genes characteristic of immune pathways between TAV.iPSC and PHH and TAV.HLC samples. (**A**) Dot plots showing hallmark pathways enriched in each sample, with dot sizes denoting the level of statistical significance and the colour gradient reflecting the average expression level of genes within each set. (**B**) Heatmap revealing expression profiles of leading-edge genes characteristic of the IL6/JAK/STAT3 signalling pathway across TAV.iPSC, TAV.HLC, PHH, and HepG2 samples.

We then used data from primary human hepatocytes (PHHs) to determine how different HLCs were to PHHs following differentiation. By performing a hypergeometric test using the hallmark gene set, we found that most immune pathways, such as the inflammatory response and interferon α/γ response, are enriched in PHHs when compared to HLCs. Other immune pathways, such as IL6/JAK/STAT3 signalling and IL2/STAT5 signalling, were found to be dysregulated in TAV.iPSCs and TAV.HLCs (Figure 7). The details of significantly upregulated genes in the IL2/STAT5 and IL6/JAK/STAT3 pathways are provided (Supplementary Table S1). Upregulated genes involved in the IL6/JAK/STAT3 pathway were only identified in PHHs. Significantly upregulated genes involved in the IL2/STAT 5 signalling pathways were identified in PHHs, TAV.HLCs, and HepG2 (Supplementary Table S1). No common genes were identified as upregulated across all three samples. While TAV.HLCs do not express all the same pathways as PHHs, this is likely due to culture-specific effects, where not all primary hepatocyte signatures are retained in vitro. There is a partial pathway activity relationship between PHHs and TAV.HLCs, as shown in Figure 3. In the IL6/JAK/STAT3 pathway, 23 genes were upregulated in PHHs and an additional 2 genes were upregulated in TAV.iPSCs. For the IL2/STAT5 signalling pathway, a total of 63 genes were found to be upregulated in TAV.HLCs (n = 22), G.PHHs (n = 21), and G.HepG2 (n = 20).

**Figure 8:**
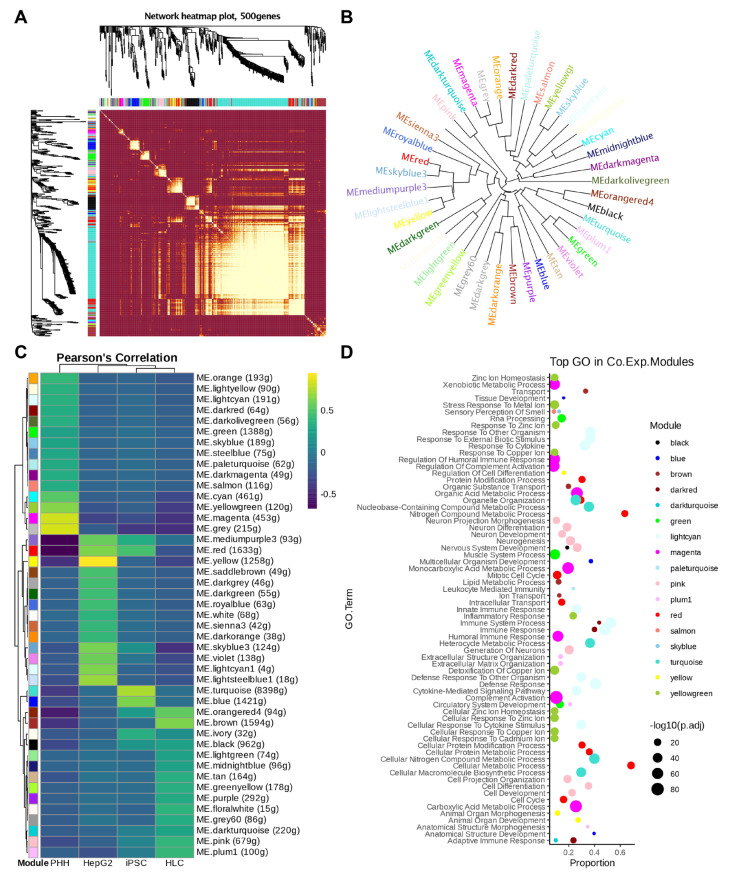
Weighted correlation network analysis (WGCNA) of iPSC, HLC, PHH, and HepG2 expression data in humans. (**A**) Heatmap showing the Topological Overlap Matrix among 500 randomly selected genes (with non-coding, etc., removed). Blocks of darker colours along the diagonal represent each module. The gene dendrogram and module assignment are also shown along the left side and top. (**B**) The dendrograms (clustering trees) of the module eigengenes in the human samples. (**C**) Pearson’s correlation of co-expression modules revealing module–sample relationships. Module eigengenes (MEs) are indicated by colours. The number of genes (g) within each module is shown in brackets. (**D**) Dot plot showing the top enriched GO terms in selected module eigengenes with the same colour codes. Bonferroni-corrected log10-converted *p* values were indicated by dot sizes.

To gain insights into the biological implications of the DEGs in each cell type, we performed weighted correlation network analysis (WGCNA), constructing an eigengene network that gives rise to 45 co-expression modules in a cell type-specific manner, and found that 14 of the modules were associated with TAV.HLCs. GO term analysis of the genes in these modules shows significant enrichment of the pathways regarding lipid metabolism, ECM organisation, and neuronal differentiation (Figure 8).

Previously, it has been shown that several liver (*ALB*, *HNF*4*A*, *HNF*1*A*, *NR*1*H*4, *ABCB*11) and intestinal genes (*CDX*2, *KLF*5, *ISX*) are co-expressed in iPSC-derived HLCs [28]. Through the analysis of gene expression datasets, we confirmed consistent findings (Supplementary Figure S1): TAV.HLCs co-express key liver markers, alongside intestinal-associated genes (*HNF*4*A*, *HNf*1*A*, *CDX*2, *KLF*5, *ISX*). This replicates similar gene expression in Liver.Norm cells, though we have only identified *HNF*4*A* and *HNF*1*A* expression in PHHs.

### 3.3. Analysis of Cancer Signatures in iPSCs or HLCs

**Figure 9:**
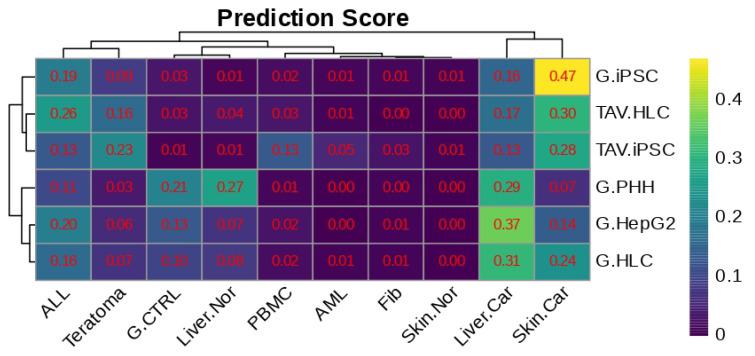
Heatmap showing similarity levels between iPSC, HLC, HepG2, and reference data by using the Random Forest algorithm. TAV.iPSCs share some common features with melanoma and teratoma, which is possibly due to the characteristics of iPSCs as proliferating cells. TAV.HLCs are less likely to share common features with hepatocellular carcinoma, judging from the lower similarity level of these cells (0.17) with liver cancer cells (Liver.Car) compared to that of the reference dataset (G.HLC) (0.31) or G.HepG2 (0.37). TAV.HLCs share a few common features with acute lymphoblastic leukaemia at a similarity level of 0.26, compared to the baseline levels of 0.13 (TAV.iPSC) and 0.19 (G.iPSC). No AML-specific features were detected in any of the TAV.iPSC and TAV.HLC samples.

Upon the molecular characterisation of HLCs, we then assessed expression profiles critical for tissue development or cancer progression. Here, we investigated similarities between HLCs and samples that characteristically recapitulate each tissue. We prepared reference datasets from (1) normal liver (Liver.Nor and G.CTRL), (2) hepatocellular carcinoma (Liver.Car), (3) normal skin (Skin.Nor), (4) melanoma (Skin.Car), (5) PBMC, (6) ALL, (7) AML, (8) teratoma, and (9) fibroblast (Fib) samples. These datasets of known phenotypes served as the input for our supervised machine learning models to be trained. We then predicted the possibilities of HLCs being classified as each of these tissue types using Random Forest. The prediction scores for each comparison are shown in the heatmap in Figure 9. This data highlighted similarities between TAV.iPSCs and TAV.HLCs with skin carcinoma. However, TAV.HLCs did not present a high prediction score with liver carcinoma, suggesting a naïve transcriptomic background.

**Figure 10:**
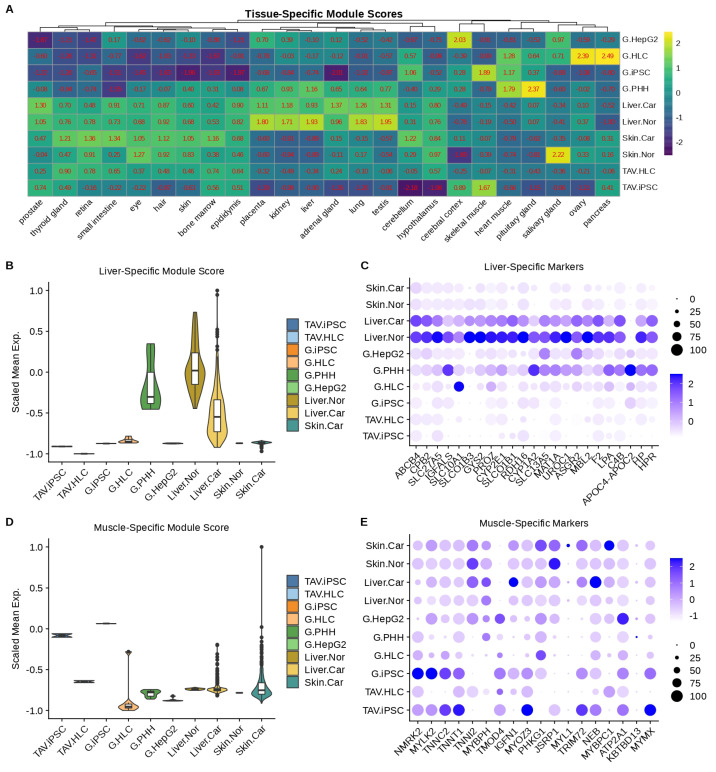
Assessment of tissue-specific gene signatures in distinct samples. (**A**) Heatmap showing the mean expression levels of the genes characteristic of each reference tissue from the HPA database (column) in each sample (row). (**B**,**D**) Mean expression levels of liver-/muscle-specific genes across different samples. (**C**,**E**) Expression profiles of each liver-/muscle-specific gene across different samples. Expression levels and percentages are indicated by colour and dot sizes, respectively.

Considering that we used all genes for prediction, which may not reflect the gene expression profiles in those cultured in vitro, we next generated distinct gene signatures characteristic of each phenotype and validated the markers by revisiting the expression profiles in the HPA datasets. We began by using the data from normal tissue from the HPA database, aiming to investigate which groups of genes for each type of human tissue could be used to characterise our TAV.iPSCs and TAV.HLCs (Figure 10). The expression profiles of liver-specific cancer genes with the greatest expression (log2 fold change > 1) and statistical significance (adjusted *p* value < 0.05) are shown in Supplementary Figure S2, indicating the similar expression of these markers in TAV.HLCs to normal liver cell gene expression. Through this analysis, we found that the control liver or skin samples were characterised by higher mean expression levels of liver- or skin-specific gene signatures. Also, the liver-specific genes were found to be highly expressed in the control samples in both the tissues and primary liver cells and expressed at lower levels in HLCs. Muscle-specific genes (negative control) were found to be highly expressed in iPSCs in both studies, but with lower expression in HLCs.

**Figure 11:**
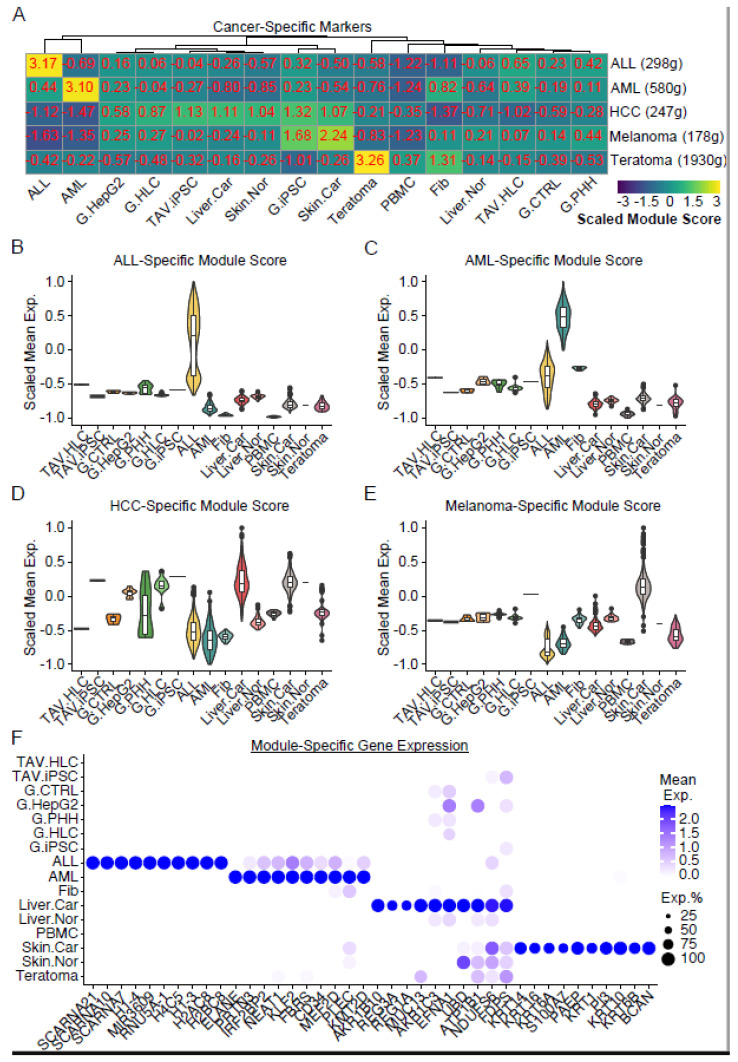
Assessment of cancer-specific gene signatures in distinct samples. (**A**) Heatmap showing the mean expression levels of the genes characteristic of each cancer. These genes were selected by analysing the top DEGs between cancer and normal tissues. The numbers of the selected genes (g) are indicated in brackets. (**B**-**E**) Violin plots showing mean expression levels of each cancer-specific gene across different samples. (**F**) Dot plot showing mean expression of top cancer-specific genes characteristic of ALL, AML, HCC, and melanoma.

Through a similar analysis, we found that cancer-specific markers were highly expressed in each of the control samples, suggesting that the gene signatures identified (Figure 11) implied cancer progression in the sample. ALL-/AML-specific gene signatures were found to be at low levels in the TAV.HLCs compared to the normal samples, such as fibroblasts or G.PHHs. Unless there is further experimental evidence that shows ALL-/AML-related characteristics in TAV.HLCs, we would expect them unlikely to exhibit leukaemic characteristics during sequencing (Figure 11). A hepatocellular carcinoma-specific gene signature was found to be at much lower levels in the TAV.HLCs compared to the reference HLCs, suggesting that the TAV.HLCs were unlikely to exhibit liver cancer characteristics during sampling (Figure 11). Finally, the expression levels of genes characteristic of melanoma or teratoma were found to be low in the TAV.HLCs.

As we identified low levels of ALL signatures in the TAV.HLCs, we investigated if this was a common trend across the iPSC-differentiated HLC sample sets. For this, we compared the ALL signature score from G.HLCs and TAV.HLCs alongside gene expression datasets in iPSC-differentiated HLCs from four separate studies (Supplementary Figure S3) [22,29,30,31,32]. All HLC samples were significantly different to the ALL samples, indicating only a partial recapitulation of ALL signatures. While the ALL signature score in the TAV.HLCs was slightly higher than that in the other samples, no significant difference was identified (*p* < 0.05, Wilcoxon test), except in Study 4, which exhibited a decreased ALL signature score. Furthermore, we analysed these samples for hepatocellular carcinoma (HCC) signature scores and identified no significant difference between the TAV.HLCs in this study and three other studies, though there was a significantly higher HCC signature score identified in two other iPSC derived HLC datasets, suggesting the usefulness of TAV.HLCs.

Due to the limitations of the HPA database, we generated cancer-specific markers for (1) ALL, (2) AML, (3) HCC, (4) melanoma, and (5) teratoma through analysing the top significantly DEG between normal and cancer samples and extracting the unique ones for downstream analysis. These data are summarised in Figure 11. This indicates that TAV.HLCs are not transcriptomically similar to HCC cells or the other cancers screened for.

## 4. Discussion

Non-cancerous human liver cell lines have been developed that have been found to be similar to PHH [33]. Human iPS cells offer an alternative source of cells for the modelling and treatment of human diseases to replace animal studies, especially mice, which have been traditionally used. These studies often cause animal suffering, generate species-specific data, are expensive to carry out, and result in unforeseen effects in vivo. iPS cells can be grown almost limitlessly and provide large amounts of material cost-effectively and relatively quickly for transcriptomics, proteomics, metabolomics, and toxicological profiling. In this study, we utilised the most advanced software packages and developed novel algorithms with optimised parameters to investigate the potential for iPSCs to provide hepatocyte-like (HLC) derivatives that could be useful as reliable surrogates for studies that are important for the liver. In this study, we found differences in the expression of proliferation and stemness markers, which have also been documented in previous studies on HLCs cultured on laminin or Matrigel^TM^. Furthermore, RIG I expression was also similar in TAV.HLCs, as has been described previously [15,34]. We also identified consistent expression in TAV.HLCs of key liver and intestinal gene markers, supporting the presence of a hybrid cellular state in line with previous observations [28].

Moderate ALL signature scores in TAV.HLCs were found, which were not significantly different to the ALL scores identified in other iPSC-derived HLC samples. While it is significantly decreased compared to the signature score in true ALL samples, this highlights the moderate cancer signature in these cells. The ALL signature score of the TAV.HLCs were found to be significantly different from the ALL signature scores identified in the HLCs in Study 4, likely due to the hyperosmolar conditions used for differentiation, suggesting specific responses due to culturing conditions. G.HLCs were analysed to more closely align with PHHs in PCA; however, the cancer prediction scores, particularly for HCC, were lower in TAV.HLCs when compared to G.HLCs, suggesting a lower carcinogenic profile in these cells and the usefulness of TAV.HLCs.

These findings support our study’s contribution to further understanding basic liver function and disease and the potential for future pharmacotoxicology studies on drug treatments and their potential side effects and even potential genotoxicity arising from the genetic modification of liver cells.

It is important to note that a major limitation to this study may be the interpretation of our data analysis originating from iPS cells and their differentiation. Batch effects should always be considered in tissue culturing and engineering. In this case, both we and the originators of the reference data grew iPSCs and their HLC-differentiated counterparts under 2D and 3D conditions, which can lead to different cellular behaviours and, therefore, different data outputs. We suspect that this is why there are differences presented between the TAV.HLC and G.HLC datasets. While TAV.HLCs and G.HLCs use similar small-molecule and growth factor cocktails (i.e., Activin A, Wnt3A, and HGF), TAV.HLCs are differentiated using a 3D spheroid culture approach using oncostatin M, EGF, bFGF, VEGF, and hydrocortisone 21-hemisuccinate, while G.HLCs are differentiated on 2D monolayers and use BMP4. Hence, we believe that cell populations should be carefully controlled, ideally with minimal heterogeneity. We chose not to synchronise cells before treatments because synchronised cells may contribute to biological variation and transcriptomic differences. Importantly, we chose to use stringent test conditions that showed that the cells exhibited markers of pluripotency at the iPSC stage, a definitive endoderm, and hepatoblasts before expressing markers true to hepatocytes by immunostaining and q-RT PCR, along with hepatocyte function, as we previously described [17].

Another consideration is our quality control for the paired-end raw sequencing data before filtering and trimming reads for our analyses. Starting with raw data, which gave rise to a count matrix for downstream analysis, and using the DESeq2 algorithm instead of a Limma trend approach could have compromised the DEG analysis. Nonetheless, directly using FPKM values for validation purposes, such as carcinogenesis assessment, should not have significantly compromised this analysis. We also used the top 5000 variable protein-coding genes for PCA to focus on these genes rather than using non-coding transcripts. If ribosomal genes were included, this could have masked many other genes with biological significance, and the PCA outcome could have been altered. We observed that most of the DEGs in the TAV.HLCs were downregulated compared to the TAV.iPSCs, which were highly proliferative. Many of these genes were found to be associated with immune response and potentially related to TNF/IFN gene expression levels. This may be due to the quiescent nature of these HLCs, as supported by previous studies, since during homeostasis, the primary function of hepatocytes is metabolic activity [34]. Although this supports the need for further validation of these findings, HLC quiescence, which occurs following differentiation, may involve IFN-gamma expression, as shown previously by the effects of IFN-gamma on hepatocyte cell cycle arrest [35].

Importantly, our analysis benefitted from statistical evidence that showed strong similarities between our TAV.HLCs and primary hepatocytes. To determine whether the transcriptome profile could be indicative of bias towards carcinogenesis, we included 1916 public samples as training datasets for prediction and focused mainly on hepatocellular carcinoma and acute lymphoblastic/myeloid leukaemia. From our interpretation of the TAV sample data, we concluded that our differentiated HLCs are unlikely to undergo carcinogenesis, although this conclusion may be limited by the selected cancers that we focused on. In future work, we would increase the training datasets, allowing for the identification of signatures of pre-malignancy.

We have shown that the transcriptome profile of iPSCs, as expected, is characteristic of cell proliferation, and it contrasts with that of the HLCs generated in this study. Most importantly, we observed our HLCs closely match the profile of primary hepatocytes and exhibit low-risk carcinogenesis. This further strengthens the hypothesis that HLCs do indeed represent primary hepatocytes and would be useful for the generation of models to be used as alternatives to using animals for drug efficacy and pharmacotoxicological testing. In conclusion, this report provides in-depth profiling of these cells and their HLC derivatives at the transcriptomic level to support their use in studies requiring genuine surrogates for PHHs isolated from living human donors.

## Figures and Tables

**Table 1:** Gene expression datasets. The gene expression datasets used in this study, derived from experimental data from TAV.iPSCs and TAV.HLCs, were compared to publicly available gene expression datasets from a range of normal and diseased phenotype cell derivatives.

#	Sample	Qty	Source	Data	Description
1	TAV.HLCs	2	In house	FPKM	Hepatocyte-like cells
2	TAV.iPSCs	2	In house	FPKM	Induced pluripotent stem cells
3	G.HepG2	7	[22]	FastQ	Liver cancer cell line
4	G.PHHs	12	[22]	FastQ	Primary human hepatocytes
5	G.HLCs	9	[22]	FastQ	Hepatocyte-like cells
6	G.iPSCs	2	[22]	FastQ	Induced pluripotent stem cells
7	G.CTRL	5	[22]	FastQ	Primary human liver tissue—healthy
8	Liver-Nor	50	TCGA database	Count	Primary human liver tissue—normal
9	Liver-Car	374	TCGA database	Count	Primary human liver tissue—cancer
10	Skin-Nor	1	TCGA database	Count	Primary human skin tissue—normal
11	Skin-Car	471	TCGA database	Count	Primary human skin tissue—cancer
12	AML	224	TCGA database	Count	Acute myeloid leukaemia
13	ALL.P1-3	679	TCGA/TARGET database	Count	Acute lymphocytic leukaemia phase 1–3
14	Teratoma	37	[23]	FPKM	Teratoma
15	Fibroblast	34	[24]	FPKM	Tissue-specific fibroblasts
16	PBMCbulk	13	[25]	TPM	Peripheral blood mononuclear cells

## Data Availability

The data presented in this study are available upon reasonable request from the corresponding author.

## Supplementary Materials

The following supporting information can be downloaded at https://www.mdpi.com/article/10.3390/cells14120925/s1: Supplementary Figure S1: Expression level assessment of liver and intestinal genes in distinct samples. This shows that TAV.HLCs co-express liver (*ALB*, *HNF4A*, *HNF1A*, *NR1H4*, *ABCB11*) and intestinal (*CDX2*, *KLF5*, *ISX*) markers, suggesting a hybrid cell state; Supplementary Figure S2: Expression level assessment of hepatocellular carcinoma-specific cancer gene signatures in distinct samples. This shows no HCC-specific markers present in TAV.HLCs, indicating a good representation of normal liver cells; Supplementary Figure S3: Expression level assessment of ALL (A) and HCC (B) signatures in liver samples. The ALL signature score was identified from transcriptomic data for PBMCs and ALL controls, exhibiting low and high scores, respectively, as expected. iPSC-derived HLCs, including G.HLCs, TAV.HLCs, and HLCs from Studies 1–4, were assessed for ALL signature score, demonstrating low but significant scores compared to the ALL control samples. The ALL signature scores for TAV.HLCs are not significantly different to the ALL signature scores in G.HLCs and Studies 1–3, with a significant decrease in the signature score highlighted in Study 4. This shows low levels of ALL signature scores in HLCs, which are comparable across many differentiation protocols. B-HCC signature scores in Liver.Nor and Liver.Car samples show a significant difference, as expected. While no significant difference is identified between TAV.HLCs and samples from Studies 1, 2, and 3, a significantly higher HCC signature score is identified in G.HLCs from Study 4. Study 1: Jo, H. Y. et al., 2020 [29]. Study 2: Nghiem-Rao, T.H. et al., (2021) [30], Study 3: Raggi, C. et al., (2022) [31], Study 4: Chui, J.S. et al., (2024) [32]; Supplementary Table S1: Differential gene expression of genes involved in the IL6/JAK/STAT3 (denoted as IL6) and IL2/STAT5 (denoted as IL2) signalling pathways across PHH, TAV.HLC, and HepG2 samples. For IL2, we highlight representative genes across PHHs, HLCs, and HepG2. For IL6, we focus on genes enriched in PHHs, as these are generally downregulated in HLCs and HepG2.
