# Supplementary material for: Transcriptomic Profiling of iPS Cell-Derived Hepatocyte-like Cells Reveals Their Close Similarity to Primary Liver Hepatocytes
Source: Cells. 2025 Jun 18;14(12):925. doi: 10.3390/cells14120925 (PMC12190443; doi:10.3390/cells14120925)
Supplement: Supplementary file 1 [file cells-14-00925-s001.zip › Supplementary table 1.pptx]

## Slide 1
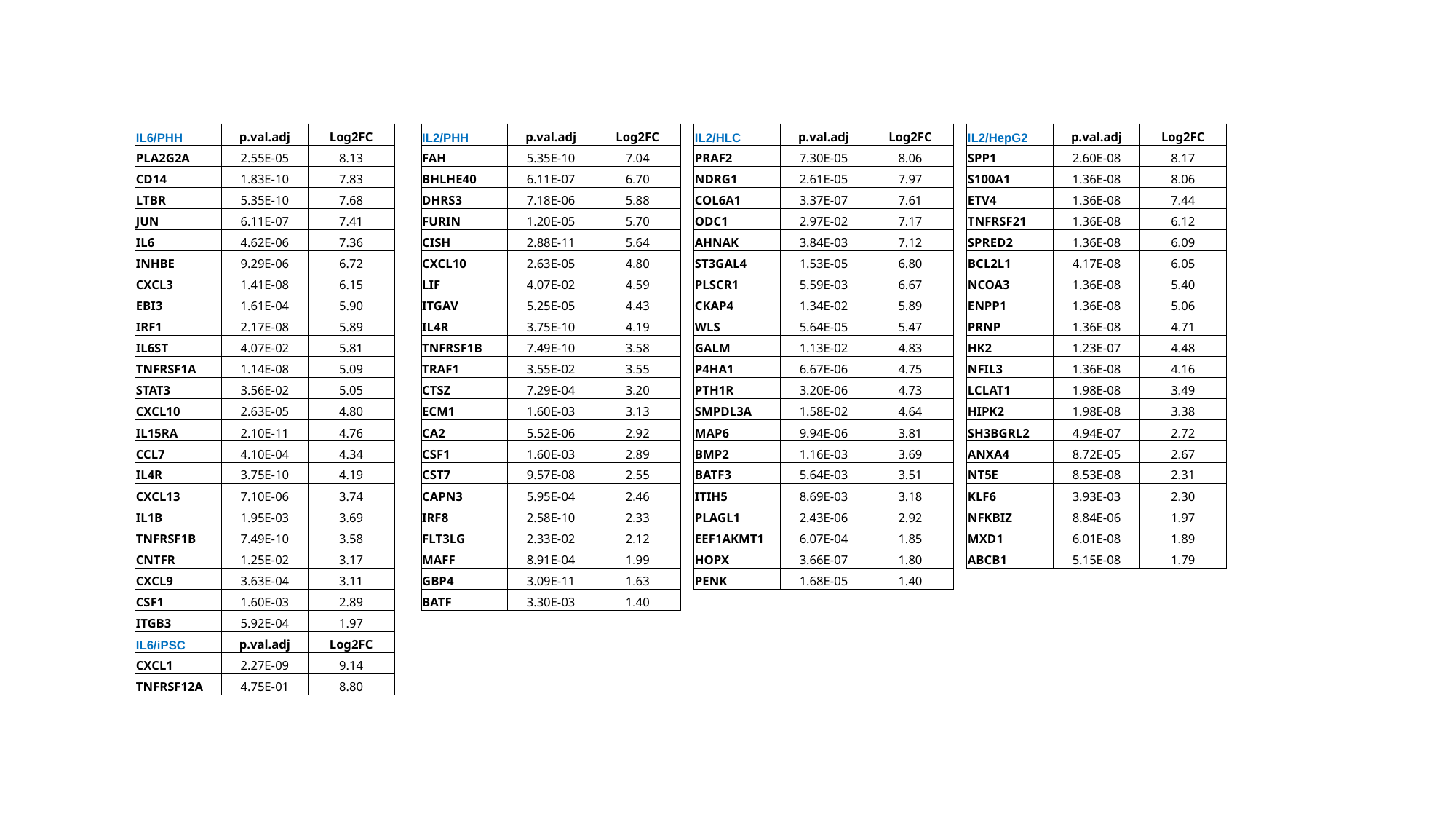

| IL6/PHH | p.val.adj | Log2FC | | IL2/PHH | p.val.adj | Log2FC | | IL2/HLC | p.val.adj | Log2FC | | IL2/HepG2 | p.val.adj | Log2FC |
| --- | --- | --- | --- | --- | --- | --- | --- | --- | --- | --- | --- | --- | --- | --- |
| PLA2G2A | 2.55E-05 | 8.13 | | FAH | 5.35E-10 | 7.04 | | PRAF2 | 7.30E-05 | 8.06 | | SPP1 | 2.60E-08 | 8.17 |
| CD14 | 1.83E-10 | 7.83 | | BHLHE40 | 6.11E-07 | 6.70 | | NDRG1 | 2.61E-05 | 7.97 | | S100A1 | 1.36E-08 | 8.06 |
| LTBR | 5.35E-10 | 7.68 | | DHRS3 | 7.18E-06 | 5.88 | | COL6A1 | 3.37E-07 | 7.61 | | ETV4 | 1.36E-08 | 7.44 |
| JUN | 6.11E-07 | 7.41 | | FURIN | 1.20E-05 | 5.70 | | ODC1 | 2.97E-02 | 7.17 | | TNFRSF21 | 1.36E-08 | 6.12 |
| IL6 | 4.62E-06 | 7.36 | | CISH | 2.88E-11 | 5.64 | | AHNAK | 3.84E-03 | 7.12 | | SPRED2 | 1.36E-08 | 6.09 |
| INHBE | 9.29E-06 | 6.72 | | CXCL10 | 2.63E-05 | 4.80 | | ST3GAL4 | 1.53E-05 | 6.80 | | BCL2L1 | 4.17E-08 | 6.05 |
| CXCL3 | 1.41E-08 | 6.15 | | LIF | 4.07E-02 | 4.59 | | PLSCR1 | 5.59E-03 | 6.67 | | NCOA3 | 1.36E-08 | 5.40 |
| EBI3 | 1.61E-04 | 5.90 | | ITGAV | 5.25E-05 | 4.43 | | CKAP4 | 1.34E-02 | 5.89 | | ENPP1 | 1.36E-08 | 5.06 |
| IRF1 | 2.17E-08 | 5.89 | | IL4R | 3.75E-10 | 4.19 | | WLS | 5.64E-05 | 5.47 | | PRNP | 1.36E-08 | 4.71 |
| IL6ST | 4.07E-02 | 5.81 | | TNFRSF1B | 7.49E-10 | 3.58 | | GALM | 1.13E-02 | 4.83 | | HK2 | 1.23E-07 | 4.48 |
| TNFRSF1A | 1.14E-08 | 5.09 | | TRAF1 | 3.55E-02 | 3.55 | | P4HA1 | 6.67E-06 | 4.75 | | NFIL3 | 1.36E-08 | 4.16 |
| STAT3 | 3.56E-02 | 5.05 | | CTSZ | 7.29E-04 | 3.20 | | PTH1R | 3.20E-06 | 4.73 | | LCLAT1 | 1.98E-08 | 3.49 |
| CXCL10 | 2.63E-05 | 4.80 | | ECM1 | 1.60E-03 | 3.13 | | SMPDL3A | 1.58E-02 | 4.64 | | HIPK2 | 1.98E-08 | 3.38 |
| IL15RA | 2.10E-11 | 4.76 | | CA2 | 5.52E-06 | 2.92 | | MAP6 | 9.94E-06 | 3.81 | | SH3BGRL2 | 4.94E-07 | 2.72 |
| CCL7 | 4.10E-04 | 4.34 | | CSF1 | 1.60E-03 | 2.89 | | BMP2 | 1.16E-03 | 3.69 | | ANXA4 | 8.72E-05 | 2.67 |
| IL4R | 3.75E-10 | 4.19 | | CST7 | 9.57E-08 | 2.55 | | BATF3 | 5.64E-03 | 3.51 | | NT5E | 8.53E-08 | 2.31 |
| CXCL13 | 7.10E-06 | 3.74 | | CAPN3 | 5.95E-04 | 2.46 | | ITIH5 | 8.69E-03 | 3.18 | | KLF6 | 3.93E-03 | 2.30 |
| IL1B | 1.95E-03 | 3.69 | | IRF8 | 2.58E-10 | 2.33 | | PLAGL1 | 2.43E-06 | 2.92 | | NFKBIZ | 8.84E-06 | 1.97 |
| TNFRSF1B | 7.49E-10 | 3.58 | | FLT3LG | 2.33E-02 | 2.12 | | EEF1AKMT1 | 6.07E-04 | 1.85 | | MXD1 | 6.01E-08 | 1.89 |
| CNTFR | 1.25E-02 | 3.17 | | MAFF | 8.91E-04 | 1.99 | | HOPX | 3.66E-07 | 1.80 | | ABCB1 | 5.15E-08 | 1.79 |
| CXCL9 | 3.63E-04 | 3.11 | | GBP4 | 3.09E-11 | 1.63 | | PENK | 1.68E-05 | 1.40 | | | | |
| CSF1 | 1.60E-03 | 2.89 | | BATF | 3.30E-03 | 1.40 | | | | | | | | |
| ITGB3 | 5.92E-04 | 1.97 | | | | | | | | | | | | |
| IL6/iPSC | p.val.adj | Log2FC | | | | | | | | | | | | |
| CXCL1 | 2.27E-09 | 9.14 | | | | | | | | | | | | |
| TNFRSF12A | 4.75E-01 | 8.80 | | | | | | | | | | | | |
