# Supplementary figures and images for: Transcriptomic Profiling of iPS Cell-Derived Hepatocyte-like Cells Reveals Their Close Similarity to Primary Liver Hepatocytes
Source: Cells. 2025 Jun 18;14(12):925. doi: 10.3390/cells14120925 (PMC12190443; doi:10.3390/cells14120925)

## Slide 1
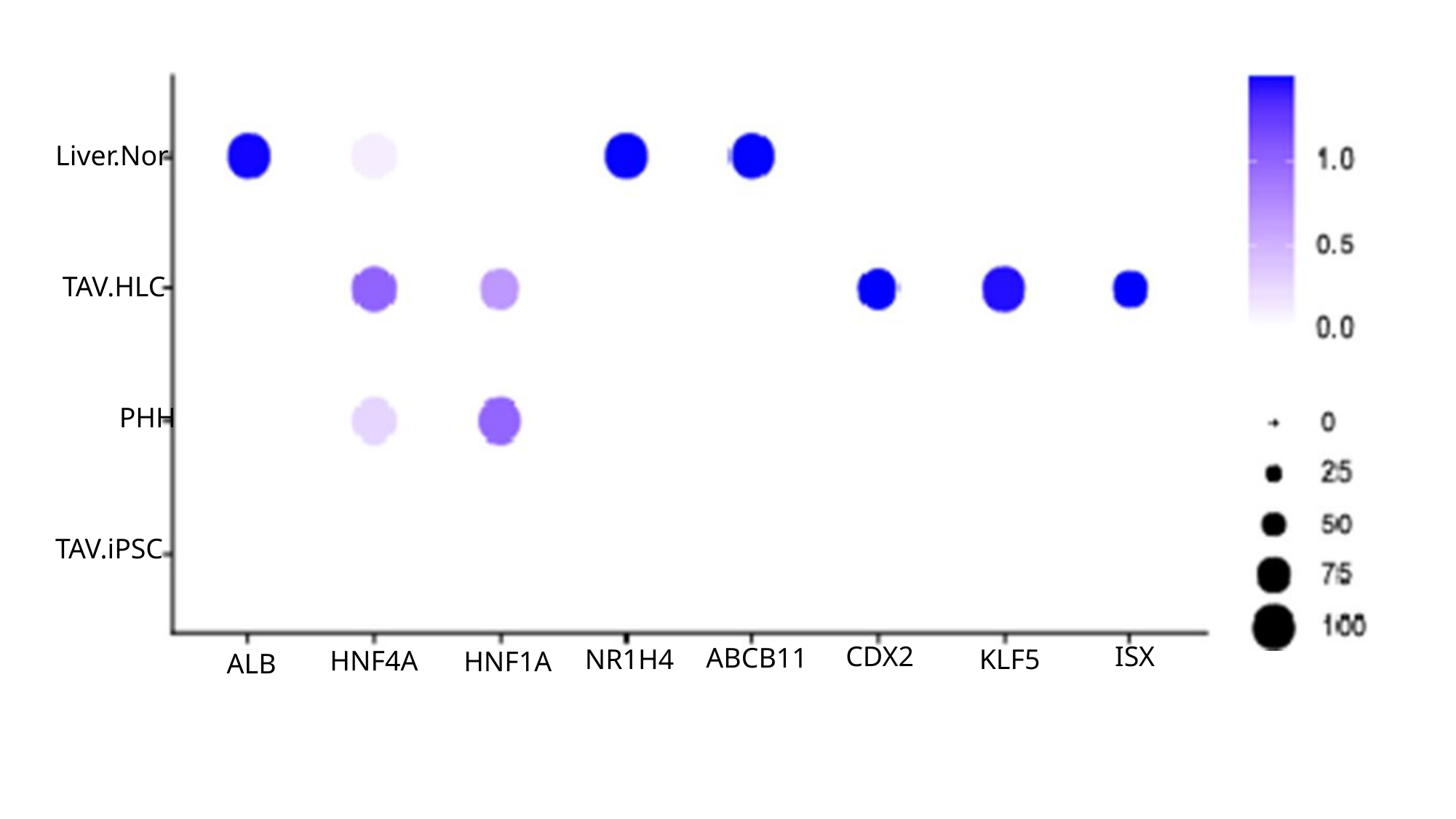

Liver.Nor
 TAV.HLC
 PHH
TAV.iPSC
CDX2
ISX
ABCB11
NR1H4
KLF5
HNF4A
HNF1A
ALB

Supplement: Supplementary file 1 [file cells-14-00925-s001.zip › Supplementary figure 1.pptx]

## Slide 1
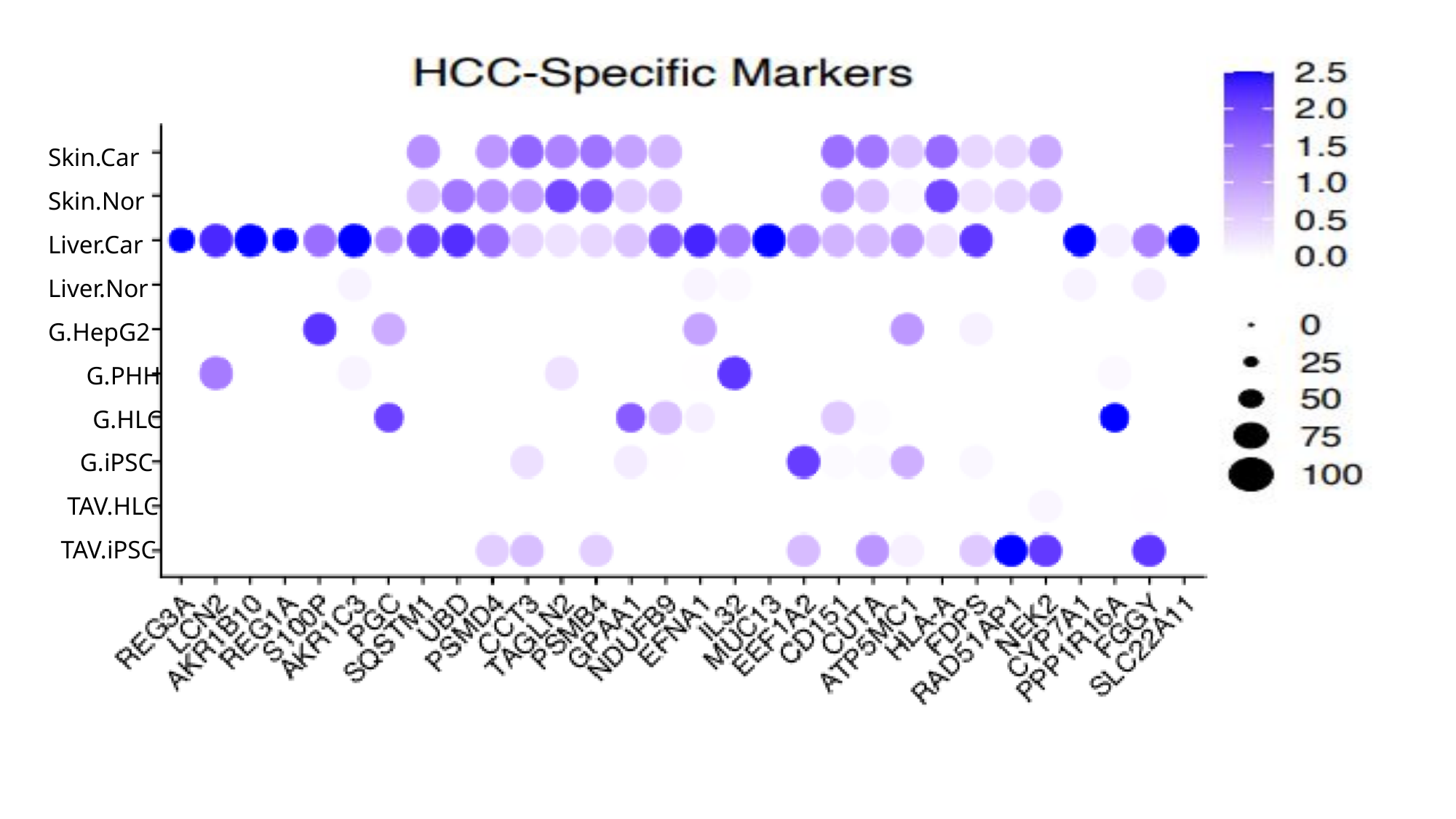

Skin.Car
Skin.Nor
Liver.Car
Liver.Nor
G.HepG2
 G.PHH
 G.HLC
 G.iPSC
 TAV.HLC
 TAV.iPSC

Supplement: Supplementary file 1 [file cells-14-00925-s001.zip › Supplementary figure 2.pptx]

## Slide 1
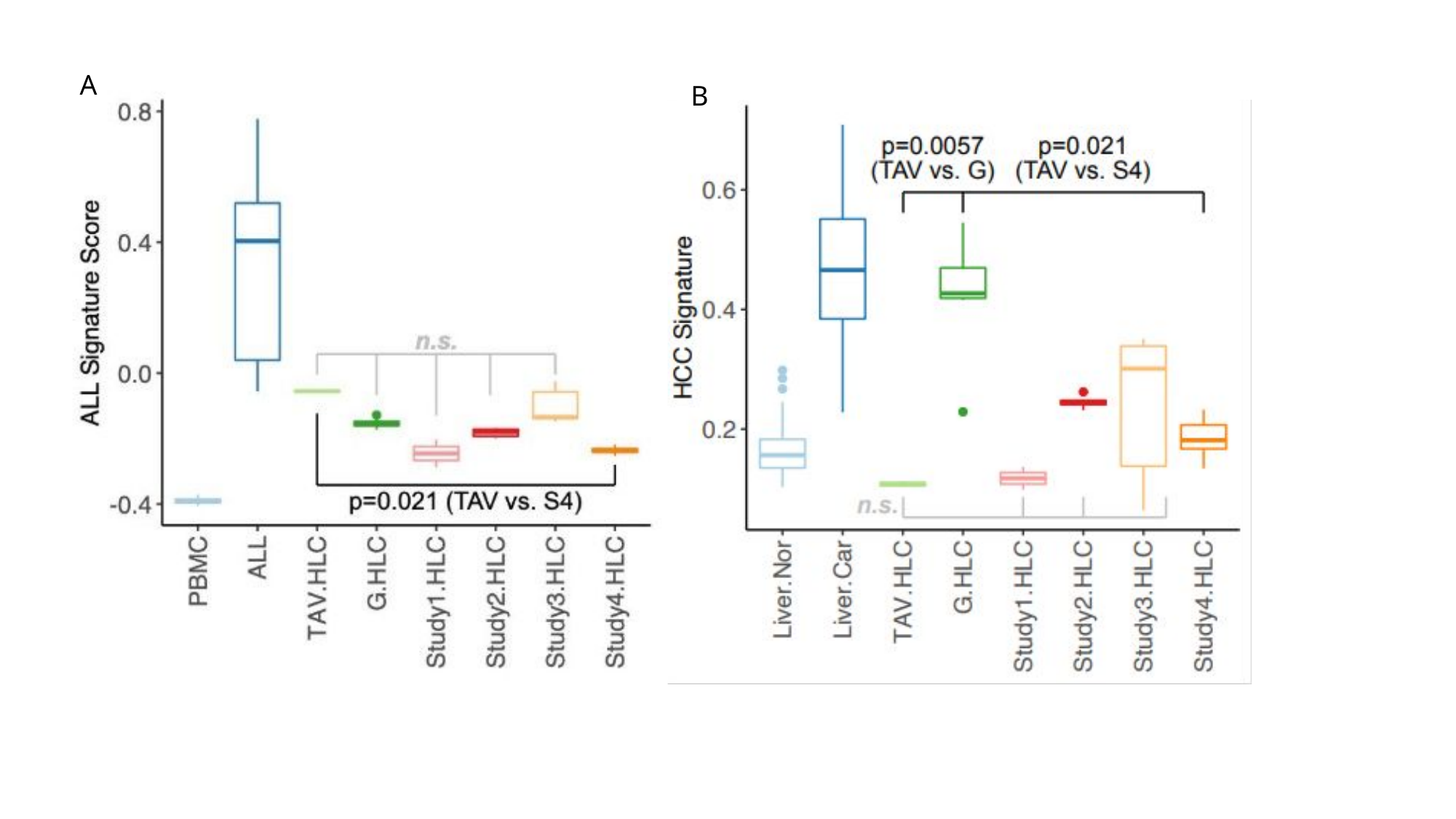

A
B

Supplement: Supplementary file 1 [file cells-14-00925-s001.zip › Supplementary figure 3.pptx]
